# Health-Promoting Additives Supplemented in Inert Microdiets for Whiteleg Shrimp (*Penaeus vannamei*) Post-Larvae: Effects on Growth, Survival, and Health Status

**DOI:** 10.3390/ani13040726

**Published:** 2023-02-17

**Authors:** André Barreto, Diogo Peixoto, Carlos Fajardo, Wilson Pinto, Rui J. M. Rocha, Luís E. C. Conceição, Benjamín Costas

**Affiliations:** 1 Riasearch Lda, Cais da Ribeira de Pardelhas, n° 21, 3870-168 Murtosa, Portugal; 2 Centro Interdisciplinar de Investigação Marinha e Ambiental (CIIMAR), Terminal de Cruzeiros do Porto de Leixões, Av. General Norton de Matos s/n, 4450-208 Matosinhos, Portugal; 3 Instituto de Ciências Biomédicas Abel Salazar (ICBAS-UP), Universidade do Porto, Rua de Jorge Viterbo Ferreira n° 228, 4050-313 Porto, Portugal; 4 Departmento de Biologia, Facultad de Ciencias del Mar y Ambientales, Instituto Universitario de Investigación Marina (INMAR), Campus de Excelencia Internacional del Mar (CEI·MAR), Universidad de Cádiz, 11510 Puerto Real, Spain; 5 Sparos Lda, Área Empresarial de Marim, Lote C, 8700-221 Olhão, Portugal

**Keywords:** *Penaeus vannamei*, whiteleg shrimp, post larvae, microdiets, dietary additives, immunostimulants, antioxidants, health status

## Abstract

**Simple Summary:**

The whiteleg shrimp (*Penaeus vannamei*) is currently the most produced species in aquaculture. However, the species larviculture is frequently associated with sub-optimal growth, high size dispersion, and low survival due to cannibalism and reduced disease resistance to pathogens. There is evidence that dietary additives can stimulate the shrimp immune system, but few studies have focused on the initial developmental stages. Therefore, this study aimed to evaluate the potential beneficial effects of several nutrients/additives (i.e., vitamins C and E, *β*-glucans, taurine, and methionine) supplemented in microdiets for whiteleg shrimp post larvae. The additives tested had no effect on growth performance and survival, but results suggest that vitamins C and E and *β*-glucans may impact the shrimp post-larvae antioxidant capacity and robustness, especially when coupled together. These findings suggest that tailored diets including these health-promoting additives may address some of the larviculture problems and may contribute to the success of whiteleg shrimp farming in the long term, affecting the downstream production of high-quality juveniles and adults.

**Abstract:**

Dietary additives have the potential to stimulate the whiteleg shrimp immune system, but information is scarce on their use in diets for larval/post-larval stages. The potential beneficial effects of vitamins C and E, *β*-glucans, taurine, and methionine were evaluated. Four experimental microdiets were tested: a positive control diet (PC); the PC with decreased levels of vitamin C and E as negative control (NC); the PC with increased taurine and methionine levels (T + M); and the PC supplemented with *β*-glucans (BG). No changes in growth performance and survival were observed. However, post-larvae shrimp fed the NC had lower relative expressions of *pen-3* than those fed the PC, suggesting that lower levels of vitamins C and E may impact the shrimp immune status. Lipid peroxidation levels dropped significantly in the BG compared to the PC, indicating that *β*-glucans improved the post-larvae antioxidant mechanisms. Furthermore, when compared with the NC diet, PL fed with BG showed significant increases in tGSH levels and in the relative expression of *crus* and *pen-3*, suggesting a synergistic effect between vitamins C and E and *β*-glucans. Amongst the additives tested, *β*-glucans seems to be the most promising even when compared to a high-quality control diet.

## 1. Introduction

The whiteleg shrimp (*Penaeus vannamei*) is currently the most representative animal species in aquaculture, constituting in 2020 a share of 4.7% in global production [1]. To meet the market demands, whiteleg shrimp larvae and post-larvae (PL) yields in hatcheries has increased intensively. However, problems in larviculture can have an enormous impact on shrimp performance in the long-term, affecting the downstream production of high-quality juveniles and adults. Initial developmental stages are frequently associated with sub-optimal growth, high size dispersion, and low survival due to cannibalism and reduced disease resistance to opportunistic pathogens. The latter results from a high dependence on optimal zootechnical conditions and nutrition, as shrimp lack an adaptive immune response and depend uniquely on their innate immune system to maintain a good health status and avoid pathogenic outbreaks that can result in disastrous consequences to production and significant economic losses [2,3,4]. Besides the fact that shrimp cannot be vaccinated due to the lack of an adaptive immune system, the use of antibiotics in the aquaculture industry is limited due to inherent food safety concerns, environmental issues, and the increased antimicrobial resistance [5]. Consequently, the use of functional dietary additives to stimulate the shrimp immune system has been studied as a prophylactic alternative and is regarded as an extremely important strategy to overcome the constraints of intensive shrimp farming. Organic acids, plant/algae extracts, nucleotides, functional amino acids, vitamins, and naturally occurring immunostimulant compounds such as *β*-glucans have been studied thoroughly in diets for fish and crustaceans, as they may improve growth performance, survival, stress, and disease resistance, as recently reviewed by Dawood et al. [6]. Their potential to be included in diets for juvenile and/or adult whiteleg shrimp has also been demonstrated to some extent by several authors [7,8,9,10,11,12,13,14,15,16,17,18]. Nevertheless, far fewer studies are available on the use of these supplements in diets for whiteleg shrimp initial developmental stages. There is evidence that vitamin C supplemented in diets for PL can be an effective antioxidant at the tissue level [19]. More recently, increases in growth performance, digestive enzymes activity, and improvement of immune condition were verified when incorporating commercial prebiotic and probiotic blends in diets for PL [20,21,22].

Hence, innovative nutritional solutions that enhance development and resistance to stress and pathogenic factors during these critical stages and thus improve shrimp quality in posterior phases of production have tremendous potential to reinforce the success of shrimp farming. Therefore, this study aimed to evaluate the effects of several health promoting nutrients/additives (i.e., vitamins C and E, *β*-glucans, taurine, and methionine) supplemented in inert microdiets on the growth performance and health status of whiteleg shrimp post larvae.

## 2. Materials and Methods

### 2.1. Dietary Treatments

Four experimental microdiets were evaluated in triplicates. A positive control diet (PC) was formulated to meet the nutritional requirements of whiteleg shrimp post larvae, containing 515 g kg^−1^ of SPAROS proprietary marine protein mix, 160 g kg^−1^ of SPAROS proprietary plant protein mix, 103 g kg^−1^ of fish protein hydrolysate, 19 g kg^−1^ of fish oil, 28 g kg^−1^ of marine phospholipids, and 57 g kg^−1^ of SPAROS proprietary vitamins and minerals premix. On the remaining treatments, three experimental variants based on the PC were used, differing only in the ingredient formulation by the following: (1) decreasing inclusion levels of the vitamins and minerals premix by 7 g kg^−1^ to reduce vitamin C and E contents in the negative control diet (NC); (2) supplementing 5 g kg^−1^ of taurine and 10 g kg^−1^ of methionine to increase the levels of both molecules in the T + M diet; and (3) supplementing the PC diet with 1 g kg^−1^ of *Saccharomyces cerevisiae β*-(1, 3)/(1, 6)-glucans (BG). The experimental diets formulation can be seen in Table 1. The proximate composition of the experimental diets was analyzed by Eurofins Food Testing Portugal following their standard procedures (Table 2).

All diets were produced at Sparos Lda facilities (Olhão, Portugal), using extrusion at low temperature as the main production process, as follows: powder ingredient mixing according to target formulation using a double-helix mixer; grinding in a micropulverizer hammer mill (SH1, Hosokawa-Alpine, Augsburg, Germany); addition of the oil fraction; humidification and agglomeration through low-temperature extrusion (Dominioni Group, Lurate Caccivio, Italy); drying of resultant pellets in a convection oven (OP 750-UF, LTE Scientifics, Oldham, UK) for 4 h at 60 °C; crumbling (Neuero Farm, Melle, Germany); and sieving to desired size ranges.

### 2.2. Shrimp Rearing and Sampling

Whiteleg shrimp post larvae (PL16), originated from Blue Genetics (La Paz, Mexico), were reared for 18 days at Riasearch Lda facilities (Murtosa, Portugal). Shrimp were randomly distributed to 12 tanks with approximately 50 L that were part of a clear water-recirculating system. Each tank was stocked with 200 individuals averaging 9 mg of wet weight. These were kept under a 12 h light:12 h dark photoperiod and were fed close to ad libitum with automatic feeders that supplied eight meals a day. Feeders were cleaned daily and charged with adjusted feed quantities based on the observation of the tanks and the presence/absence of remnants from the previous day. Feed size was 400–600 µm for the first week and 600–800 µm for the remaining feeding period. Water temperature was maintained at 28.8 ± 0.3 °C, dissolved oxygen concentration at 7.5 ± 0.4 mg L^−1^, salinity at 20.3 ± 1.2, pH at 7.96 ± 0.1, NH_3_ at 0.0 ± 0.0 mg L^−1^, and NO_2_ at 0.36 ± 0.3 mg L^−1^.

At the start of the trial, a total of 60 shrimp from the initial stock were randomly selected and group weighed for initial wet weight determination. At the end of the experiment, all shrimp were weighed in groups of 20 individuals for the final wet weight determination of each tank. Additionally, 40 shrimp were randomly selected from each tank for oxidative stress and immune parameters analysis and 10 shrimp for analysis of gene expression. Shrimp were fasted for 12 h prior to samplings to ensure their guts were empty at collection. Shrimp sampled for oxidative stress and immune parameters were stored at −80 °C for subsequent analysis. Shrimp sampled for molecular biology analysis were kept in RNAlater (Sigma, St. Louis, MO, USA) at >1:5 volume ratio, at 4 °C, for 24 h prior to being stored at −20 °C. Relative growth rate (RGR), feed conversion ratio (FCR), and survival for each treatment were assessed at the end of the experiment.

### 2.3. Oxidative Stress and Immunity-Related Biomarkers

#### 2.3.1. Sample Preparation

A total of 40 whole whiteleg shrimp post larvae from each tank sampled at end of the trial were weighed and homogenized in quadruple groups of 10 individuals for oxidative stress and immune parameters analysis. Potassium phosphate buffer (0.1 M) was added to each group in a 1/10 (*w*/*v*) proportion followed by homogenization using a high-performance dispersing instrument (SilentCrusher M, Heidolph Instruments, Schwabach, Germany). An aliquot for lipid peroxidation (LPO) with butylated hydroxytoluene was reserved prior to centrifugation. After centrifugation (5500 rpm for 20 min), sample supernatant was collected and distributed in separate aliquots for oxidative stress parameters and immune parameters. The remaining 10 shrimp sampled for molecular biology analysis were homogenized in NZYol (Nzytech, *w*/*v* proportion according to the manufacturer’s instructions) using a Precellys 24 tissue homogenizer (Bertin instruments, Montigny-le-Bretonneux, France).

#### 2.3.2. Determination of Oxidative Stress Biomarkers

Catalase (CAT), lipid peroxidation (LPO), and total glutathione (tGSH) activities as well as total proteins content were determined in the homogenized samples. Total proteins were measured by using PierceTM BCA Protein Assay Kit, as described by Costas et al. [23]. Samples were diluted in K-phosphate buffer (0.1 M; pH 7.4), and bovine serum albumin (BSA, 2 mg mL^−1^) was used as standard. Afterwards, 25 μL of each diluted sample and standards were plated in triplicate and read at 562 nm in a Synergy HT microplate reader. Results were calculated using a standard curve and expressed as mg mL^−1^.

CAT activity levels were determined measuring the decrease of hydrogen peroxide (H_2_O_2_ 30%, Sigma) concentration as described by Clairborne [24]. The reaction mixture was composed of K-phosphate buffer (0.05 M pH 7.0) and H_2_O_2_ (30%) as substrate, and 10 µL of homogenate sample was added to the reaction mixture, effecting a total volume of 300 µL. Absorbance was read at 240 nm in UV microplates for 2 min (1 reading every 15 s) in a Synergy HT microplate reader and results expressed as enzyme units per milligram of total protein (U mg^−1^ protein). One enzyme unit is the amount of enzyme needed to catalyze one micromole of substrate per minute.

Endogenous LPO was assessed by measuring thiobarbituric acid-reactive substances (TBARS), preventing artefactual lipid oxidation by adding butylhydroxytoluene (4%; Sigma) [25]. Homogenate samples incubated for 60 min at 100 °C with a 100 µL of trichloroacetic acid 100% solution and 1 mL of 2-thiobarbituric acid 0.73% (Sigma), trizma hydrochloride (Sigma), and diethylenetriaminepentaacetic acid (Fluka) solution in polystyrene microtubes. Afterwards, these were centrifuged for 5 min at 11,500 rpm, and supernatant (200 µL) was added to the microplate wells. Absorbance was read at 535 nm and results expressed as nmol g wt^−1^.

Total glutathione content in post-larvae homogenate samples was measured based on the oxidation of glutathione by 5,5′-dithiobis-(2-nitrobenzoic acid) (DTNB; Sigma) as described by Rodrigues et al. [26]. Samples were diluted in K-phosphate buffer (0.1 M pH 7.4) to obtain 0.7 mg mL^−1^ of protein. Thereafter, 50 µL of each diluted sample was added to microplate wells, followed by the addition of 250 µL of a reaction solution composed by DTNB, K-phosphate buffer (0.1 M, pH 7.4), NADPH (ß-Nicotanimide adenine dinucleotide 2′-phosphate reduced tetrasodium salt; Alpha Aesar), and glutathione reductase (Sigma). Absorbance was read at 412 nm for 3 min (1 reading every 20 s) in a Synergy HT microplate reader and results expressed as nmol mg of protein^−1^.

#### 2.3.3. Analysis of Immune Parameters

Lysozyme, pro-phenoloxidase, and bactericidal activities were determined in the homogenized samples. Lysozyme activity was measured using a turbidimetric assay as described by Costas et al. [27]. Briefly, a solution of *Micrococcus lysodeikticus* (0.25 mg mL^−1^, 0.05 M sodium phosphate buffer, pH 6.2) was prepared and 40 μL of homogenized samples, and 130 μL of this suspension were added to a microplate, effecting a final volume of 170 μL. The reaction was carried out at 25 °C, and absorbance (450 nm) measured after 0.5 and 30 min in a Synergy HT microplate reader. Lyophilized hen egg white lysozyme (Sigma) was diluted in sodium phosphate buffer (0.05 M, pH 6.2) and used to develop a standard curve. The amount of lysozyme in the sample was calculated using a standard curve. Lysozyme was expressed as µg mg protein^−1^.

Pro-phenoloxidase activity was measured spectrophotometrically using L-DOPA (L-3,4- dihydroxyphenylalanine) as substrate and trypsin (Sigma) as activator following the method described by Ji et al. [28] with modifications. Homogenate samples of 50 μL were diluted in 100 μL of trypsin solution (0.1% in cacodylate solution) in a 96-well microplate and incubated for 30 min at room temperature. Afterwards, 100 μL L-DOPA solution (0.3% in cacodylate solution) was added. The absorbance was measured every minute during 5 min at 490 nm using a Synergy HT microplate reader. Results were calculated using the Beer–Lambert law using the molar extinction coefficient of the L-DOPA (3700). Results were expressed as units of pro-phenoloxidase mL^−1^ of sample.

*Vibrio harveyi* was used in the bactericidal activity assay. Exponentially growing bacteria were resuspended in sterile HBSS and adjusted to 3.1 × 10^9^ cfu mL^−1^. Plating serial dilutions of the suspensions onto TSA-2 plates and counting the number of cfu following incubation at 22 °C confirmed bacterial concentration of the inoculum. Homogenized samples’ bactericidal activity was then determined following the method described by Machado et al. [29]. Briefly, 60 µL of homogenized samples were added to a U-shaped 96-well plate. HBSS was added to some wells instead of homogenized samples and served as positive control. To each well, 20 μL of *V. harveyi* (3.1 × 10^9^ cfu mL^−1^) were added, and the plate was incubated for 2.5 h at 25 °C. To each well, 25 μL of 3-(4, 5 dimethyl-2-yl)-2, 5-diphenyl tetrazolium bromide (1 mg mL^−1^; Sigma) was added and incubated for 10 min at 25 °C to allow the formation of formazan. Plates were then centrifuged at 2000× *g* for 10 min, and the precipitate was dissolved in 200 μL of dimethyl sulfoxide (Sigma). The absorbance of the dissolved formazan was measured at 560 nm. Bactericidal activity was expressed as percentage calculated from the difference between the surviving bacteria compared to the number of bacteria from positive controls (100%).

#### 2.3.4. Gene Expression Analysis

Extraction of RNA was performed using the NZY total RNA isolation kit (NZYTech, Lisboa, Portugal) according to the manufacturer’s instructions. RNA concentration and purity was analyzed by spectrophotometry using DeNovix DS-11 FX (Wilmington, NC, USA). RNA concentration varied from 123.9 to 2180.7 ng µL^−1^ and 260:280 ratios between 1.99 and 2.17, respectively. The integrity of the RNA samples was verified through a 2% agarose gel. The cDNA was obtained using the NZY first-strand cDNA synthesis kit (NZYTech). This step was used to standardize the concentration of the samples. Reverse transcription was carried out in a Veriti DX 96-well thermal cycler (Applied Biosystems, Waltham, MA, USA), using 4.4 μL of diluted cDNA (20 ng µL^−1^) mixed with 5 μL of NZYSpeedy qPCR Green Master Mix^®^ (NZYTech) and 0.3 μL (10 μM) of each specific primer in a final volume of 10 μL. Real-time quantitative PCR was performed, in duplicate for each sample, using a CFX384 Touch Real-Time PCR Detection System (Biorad, Hercules, CA, USA). Nine genes were selected and analyzed due to their role in the immune response. Primer efficiency was tested for each gene (Table 3). Cycling conditions were the same between the different genes, consisting of one cycle of 95 °C for 10 min, followed by 40 cycles of 2 steps of 95 °C for 15 s and 62 °C for 1 min, with a final cycle at 95 °C for 1 min, followed by 35 s at 62 °C and ending at 95 °C for 0.5 s. The Pfaffl method [30] was used to perform gene expression analyses, and target genes were normalized using *bactn* and *rpl-8* as housekeeping.

### 2.4. Data Analysis

Relative growth rate (RGR, % weight day^−1^) was calculated as follows: RGR = (e^g^ − 1) × 100, where e = exponential and g = (lnWf − lnWi) × t^–1^. Wf and Wi correspond to the final and initial weights, respectively. Feed conversion ratio (FCR) was calculated as follows: FCR *=* (Fi/Wg), where Fi corresponds to feed given (g) and Wg to the mean weight gain (g). Survival was expressed as percentage and calculated as follows: S = (Lf/Li) × 100, where Li and Lf correspond to the initial and final number of post larvae in the tanks, respectively. Differences in growth performance, FCR, survival, oxidative stress, immune condition, and gene expression between dietary treatments were evaluated using one-way ANOVAs, followed by Tukey multiple comparison tests. Kruskal–Wallis one way analysis of variance tests followed by Wilcoxon pairwise comparison tests were used when data did not comply with the one-way ANOVA’s assumptions. Results were expressed as means ± standard deviation (SD). In results expressed as percentage, an arcsine transformation was performed prior to any statistical test: T = ASIN (SQRT (value/100)). The significance level considered was *p* < 0.05 for all tests performed.

All activities were undertaken within the clear boundaries of national and EU legal frameworks directed by qualified scientists/technicians and conducted according to the European guidelines on the protection of animals used for scientific purposes (Directive 2010/63/UE of the European Parliament and on the European Union Council) and under strict monitoring and control of DGAV—(Direção Geral de Alimentação e Veterinária), Animal Welfare Division, which is the competent authority responsible for implementing the legislation on the “protection of animals used for scientific purposes”.

## 3. Results

### 3.1. Growth Performance

No significant differences in growth performance and survival were observed among dietary treatments. Final wet weight averaged around 100 mg, RGR values 15% day^−1^, FCR was close to 1, and survival ranged between 86 to 88% for all treatments (Table 4).

### 3.2. Oxidative Stress and Immune Status Related Biomarkers

Regarding the oxidative stress parameters measured, CAT levels were similar, with no significant differences being detected across treatments; LPO levels were significantly lower in shrimp PL fed the BG dietary treatment than those fed the PC diet, with no significant differences between the remaining treatments; tGSH levels were significantly higher in shrimp PL fed the BG treatment than in their counterparts fed the NC diet, with no significant differences between the remaining treatments. As for the immune condition, no significant differences between treatments were observed regarding the parameters measured (Table 5).

### 3.3. Gene Expression Analysis

The normalized relative mRNA expression of the PvHm117 crustin P gene decreased significantly in shrimp PL fed the NC diet compared to those fed the T + M and BG dietary treatments. Similarly, the penaeidin-3a mRNA expression level decreased significantly in shrimp PL fed the NC diet compared to their counterparts fed the PC and BG dietary treatments. Hemocyanin transcripts increased significantly in shrimp PL fed the NC diet compared to PL fed the T + M dietary treatment. As for the normalized relative mRNA expression of the remaining genes, no significant differences between treatments were observed (Table 6).

## 4. Discussion

This study aimed at evaluating the potential health-promoting effects of including several dietary supplements in inert microdiets for whiteleg shrimp PL. Vitamin C and E, methionine, taurine, and *β*-glucans were selected for this purpose since their potential ability to enhance the health status of whiteleg shrimp in the initial stages of development is still promising but yet to be experimentally validated. A control diet formulated to fulfil the nutritional requirements of whiteleg shrimp PL was used as positive control, and the remaining experimental diets were based on it, differing only in the reduction or addition of the previously mentioned nutrients. In overall, the formulation changes in the diets did not compromise their adequacy, as good growth performances and survival results were obtained in all experimental treatments, also revealing that good zootechnical conditions were maintained during the trial. Growth results were similar to those reported by Wang et al. when using graded levels of *Schizochytrium* meal [31] and as a replacement of fish oil [32] in practical diets for whitleg shrimp PL, but survival results were considerably inferior in those studies (40.3–44.5% and 42.7–45.6%, respectively) than in the current trial.

Increasing the vitamin C and E supplementation levels in the PC dietary treatment did not produce any changes in growth performance and survival when compared with the NC, suggesting that the levels of these vitamins present in the NC diet still allowed the shrimp post larvae to maintain an adequate development. Like other vitamins, vitamin C and E are essential nutrients, as animals are unable to synthesize sufficient amounts to meet their physiological needs, and a deficient supply in the diet often results in poor growth, possibly leading to severe health issues and even compromising survival [6,33]. Additionally, no changes were verified in the activity levels among most of the immune and antioxidant parameters measured in this study when increasing the levels of these vitamins in the diets. Accordingly, other studies reported thresholds in inclusion levels for these vitamins in diets for whiteleg shrimp juveniles at which their beneficial effects did not increase after a certain incorporation percentage [7,14]. The only significant dissimilarity detected was the lower relative expression of *pen-3* in shrimp fed the NC diet compared to those fed the PC diet, suggesting that lower levels of these vitamins may impact the shrimp immune status. Penaeidins, a key group of antimicrobial peptides in penaeid shrimp, have antibacterial and antifungal activities, which are particularly effective against Gram+ bacteria and filamentous fungi [34,35]. These findings indicate that higher supplementation levels of vitamins C and E in microdiets for whiteleg shrimp PL did not directly enhance growth and survival in the current study but may have improved their robustness. Although not confirmed in the current experiment, this may be an indication that shrimp may have a higher survival capacity in the long term and particularly in a potentially challenging husbandry situation. Therefore, inert diets with adequate levels of vitamins C and E can be vital during critical stages of production, particularly in farms where a nursery system is employed (intermediate step between the early PL stage and the grow out phase), in which PL are kept at extremely high stocking densities that can induce stress and vulnerability to opportunistic pathogens [36].

The supplementation of methionine to balance the nutritional profiles of aquafeeds rich in plant-based proteins has become a common practice [16,37]. Traditionally, methionine supply was ensured by fish meal, but continuous efforts are underway to reduce the industry dependence on this ingredient and replace it by plant-based proteins, where methionine and lysine are generally low in the amino acid profile. It has been shown by several authors that when dietary requirements are not met, generally, when low-fish-meal diets are concomitantly used, growth performances and survival of whiteleg shrimp can be affected [13,16,38,39,40]. Additionally, methionine has a recognized role in the immune system and has recently been used to improve the antioxidant capacity, innate immune response, and/or disease resistance of whiteleg shrimp juveniles [16,18,41]. Besides that, methionine is also a precursor for taurine. The supplementation of this nutrient in diets for whiteleg shrimp is also recommended since it can provide beneficial effects on their growth and immune response [12,17]. Yet, the evaluation of the supplementation of both molecules in diets for the initial developmental stages of shrimp is still necessary. In this study, whiteleg shrimp PL fed with the T + M diet showed similar growth performances, survival, oxidative status, and immune condition to those fed the PC dietary treatment, suggesting that the ingredient formulation of the control diet was capable of covering the shrimp PL requirements for taurine and methionine, and no extra benefits were obtained through the supplementation of these amino acids. These results can probably be explained by the fact that the PC was a high-quality diet with considerable levels of protein of marine origin. However, shrimp PL fed the T + M diet showed significantly higher relative expressions of *crus* and significantly lower *hmc* transcripts when compared with those fed the NC dietary treatment. Both PvHm117 crustin P and hemocyanin are associated with important broad-spectrum antimicrobial peptides involved in the first line of the shrimp defense [42,43,44]. These results could be considered contradictory, as it would be expected that variations in the expressions of both genes would follow the same trend. Still, it is important to bear in mind that hemocyanin is a multifunctional protein involved in several physiological processes beyond innate immunity, such as oxygen transport, protein storage, molt cycle, exoskeleton formation, and osmoregulation [45,46]. Concomitantly, taurine is also one of the main organic osmolytes in osmoregulation for decapods [47,48], and it has been shown that increases in dietary taurine inclusion levels increases the molecule contents in different tissues and hemolymph of whiteleg shrimp [17]. Therefore, the hemocyanin levels needed to maintain osmolality were probably lower in shrimp fed the T + M diet, which may have caused a downregulation of the *hmc* gene. Still, it should be noted that the analysis only focused on measuring mRNA transcripts of *hmc* and not the taurine molecule levels. Therefore, to better understand these interactions and clarify if the supplementation of taurine and methionine in diets for whiteleg shrimp PL is beneficial when lower vitamin C and E inclusion levels are used, further studies should be conducted.

The Inclusion of *β*-glucans in the diets did not significantly affect the shrimp PL growth performance and survival. Still, shrimp PL fed the BG diet tended to grow less and achieved final weights around 15% lower than those fed the PC, although it was not supported by the statistical analysis. Nonetheless, lipid peroxidation levels dropped significantly in shrimp PL fed the BG dietary treatment compared to those fed the PC diet, suggesting that *β*-glucans improved the antioxidant mechanisms of the animals. In fact, the immunostimulatory and antioxidant-boosting properties of *β*-glucans as aquafeed additives have been reported for several species, as recently reviewed by Pogue et al. [49]. These can be tremendously valuable in the larval/PL stages, where shrimp undergo extremely fast development, as accelerated growth is likely to produce excess reactive oxygen species that can result in oxidative stress, damaging key physiological structures [50]. Although there are reports of the enhancement of whiteleg shrimp disease resistance through the dietary supplementation of *β*-glucans [51,52,53], in the present study, no significant improvements in immune condition were observed in the shrimp PL fed the BG diet compared with those fed the PC diet. Bai et al. [54] suggested that discontinuous feeding, changing between a basal diet and one with the inclusion of *β*-glucans, is the most suitable strategy to enhance the immunity of whiteleg shrimp, as continuous feeding for long periods of time with the supplemented diet can cause immune fatigue, mitigating the beneficial effects provided in the short term. Considering this hypothesis, the BG diet potential to improve the whiteleg shrimp PL immunity could have been clearer if a different feeding strategy had been employed. When compared with the NC diet, PL fed with BG showed significant increases in tGSH levels as well as in the relative expression of *crus* and *pen-3*. This suggests that the *β*-glucans supplementation coupled with higher levels of vitamin C and E can boost the antioxidant capacity and immune status of whiteleg shrimp PL. In fact, Wu et al. [14] proposed that there is an interaction between *β*-glucans and vitamin C that is capable of increasing the nonspecific immune response of the whiteleg shrimp. The results obtained in that study corroborate this hypothesis, as the addition of *β*-glucans to the PC diet amplified the differences in the shrimp’s immune condition and antioxidant capacity relative to the NC diet.

## 5. Conclusions

In conclusion, the results obtained in this study suggest that although no improvements in growth performances and survival were observed at the end of the experimental period, all dietary additives tested have the potential to add value to inert microdiets for whiteleg shrimp PL. Benefits to the antioxidant capacity and robustness of the shrimp PL were clearer when the vitamin C and E levels were higher than those used in the NC, similar to those used in the PC. However, the control diet can be considered a premium option, and it should be expected that the positive effects provided by these supplements are augmented when incorporated into more economical alternatives. Amongst the additives tested, the inclusion of *β*-glucans in the diets seems to be the most promising, as it reduced lipid peroxidation in the shrimp PL even when compared to a high-quality control diet. When compared to the NC, the interaction between the supplementation of *β*-glucans and higher levels of vitamins C and E also seems beneficial to the antioxidant capacity of whiteleg shrimp PL.

## Figures and Tables

**Table 1:** Dietary formulation of the experimental diets used to culture whiteleg shrimp (*P. vannamei*) PL for 18 days.

Ingredients (g kg^−1^)	NC	PC	T + M	BG
Marine protein mix ^1^	515	515	510	515
Fish protein hydrolysate ^2^	103	103	103	103
Plant protein mix ^3^	160	160	160	160
Cellulose ^4^	17	10	0	9
Fish oil ^5^	19	19	19	19
Marine phospholipids ^6^	28	28	28	28
Lecithin ^7^	56	56	56	56
Vitamins and minerals ^8^	50	57	57	57
Cholesterol ^9^	10	10	10	10
Antioxidant ^10^	4	4	4	4
Monoammonium phosphate ^11^	38	38	38	38
*β*-(1, 3)/(1, 6)-glucans ^12^	0	0	0	1
DL-Methionine ^13^	0	0	5	0
Taurine ^14^	0	0	10	0
^1^ Proprietary product for shrimp: 37% crude protein, 5% crude fat—SPAROS, Portugal				
^2^ Sopropêche, France				
^3^ Proprietary product for shrimp: 13% crude protein, 1% crude fat—SPAROS, Portugal				
^4^ Disproquimica, Portugal				
^5^ Sopropêche, France				
^6^ Triple nine, Denmark				
^7^ Lecico, Germany				
^8^ Proprietary premixes/products for shrimp—SPAROS, Portugal				
^9^ Carbogen, The Netherlands				
^10^ Kemin, Italy				
^11^ Timab Iberica, Spain				
^12^ MacroGard—Orffa, The Netherlands				
^13^ Premix—Especialidades Agricolas e Pecuárias Lda, Portugal				
^14^ Proprietary product for marine fish and shrimp—SPAROS, Portugal				

**Table 2:** Proximate composition of the experimental diets used to culture whiteleg shrimp (*P. vannamei*) PL for 18 days.

	NC	PC	T + M	BG
Dry matter (DM, %)	94.0 ± 0.5	93.7 ± 0.5	94.3 ± 0.5	94.3 ± 0.5
Crude protein (% DM)	66.2 ± 1.7	66.4 ± 1.7	67.4 ± 1.7	66.1 ± 1.7
Crude fat (% DM)	15.9 ± 1.0	16.2 ± 1.0	16.3 ± 1.0	15.6 ± 1.0
Fiber (% DM)	1.4 ± 0.7	1.2 ± 0.7	1.1 ± 0.7	1.2 ± 0.7
Ash (% DM)	11.4 ± 0.4	11.7 ± 0.4	11.7 ± 0.4	11.8 ± 0.4
Phosphorous (% DM)	1.9 ± 0.4	2.0 ± 0.4	1.9 ± 0.5	2.0 ± 0.4
Energy (MJ/Kg DM)	23.0 ± 0.0	23.0 ± 0.0	23.1 ± 0.0	22.9 ± 0.0
Vitamin C (mg/kg DM)	159.6 ± 0.0	2027.7 ± 0.0	2014.8 ± 0.0	2014.8 ± 0.0
Vitamin E (mg/kg DM)	42.6 ± 0.0	1067.2 ± 0.0	1060.4 ± 0.0	1060.4 ± 0.0
Taurine (g/100 g DM)	0.31 ± 0.3	0.31 ± 0.3	0.94 ± 0.9	0.31 ± 0.3
Methionine (g/100 g DM)	1.5 ± 0.2	1.5 ± 0.2	2.0 ± 0.3	0.6 ± 0.1

Results expressed as mean ± standard deviation (*n* = 2 experimental units).

**Table 3:** Selected genes and specific primers used to evaluate the immune status of whiteleg shrimp (*P. vannamei*) PL at the end of the experimental period.

Gene	Acronym	Efficiency (%)	Annealing Temperature (°C)	Accession n°	Amplicon Length (bp)	Primer Sequence (5′-3′)
Cytoplasmic-type actin 4	*bactn*	83.2	62	MF627841.1	260	F: CACGAGACCACCTACAACTCCATC R: TCCTGCTTGCTGATCCACATCTG
Ribosomal protein L8	*rpl-8*	90	62	DQ316258.1	219	F: AGCCAAGCAAGATGGGTCG R: TGTAACGATAAGGGTCACGGAAG
PvHm117 crustin P	*crus*	81	62	AY488497.1	109	F: GAAACCACCACCAACACCTACTCC R: TCTGTGCGGCCTCTTTACGG
Penaeidin-3a	*pen-3*	86.1	62	Y14926.1	137	F: ATACCCAGGCCACCACCCTT R: TGACAGCAACGCCCTAACC
Hemocyanin	*hmc*	92	62	KY695246.1	124	F: GTCTTAGTGGTTCTTGGGCTTGTC R: GGTCTCCGTCCTGAATGTCTCC
Lysozyme C-like	*lys*	73	62	XM_027352857	82	F: CGGGAAAGGCTATTCTGCCT R: CCAGCACTCTGCCATGTACT
C-type lectin 2-like	*lect*	83	62	DQ858899.2	138	F: GCTTCTGTTGGTGCTGTTGGC R: GTTCCCTTCCCGTATGTGGC
Thioredoxin 1	*trd*	85.3	62	EU499301.1	116	F: TTAACGAGGCTGGAAACA R: AACGACATCGCTCATAGA
Glutathione transferase	*gst*	99	62	AY573381	146	F: AAGATAACGCAGAGCAAGG R: TCGTAGGTGACGGTAAAGA
Glutathione peroxidase	*gpx*	86.6	62	XM_027372127.1	117	F: AGGGACTTCCACCAGATG R: CAACAACTCCCCTTCGGTA
Caspase 3	*casp-3*	93.6	62	KC660103.1	182	F: ACATTTCTGGGCGGAACACC R: GTGACACCCGTGCTTGTACA

**Table 4:** Initial and final weight, relative growth rate (RGR), feed conversation ratio (FCR), and survival of whiteleg shrimp (*P. vannamei*) PL during the experimental period.

	NC	PC	T + M	BG
Initial weight (mg)	8.8 ± 0.0			
Final weight (mg)	110.8 ± 19.3	110.8 ± 18.4	114.0 ± 9.5	94.4 ± 9.2
RGR (% day^−1^)	15.0 ± 1.1	15.0 ± 1.1	15.3 ± 0.5	14.0 ± 0.6
FCR	0.9 ± 0.0	0.9 ± 0.2	0.9 ± 0.1	1.0 ± 0.2
Survival (%)	87.0 ± 6.6	86.2 ± 7.6	85.5 ± 6.1	87.5 ± 5.0

Results expressed as mean ± standard deviation. For initial weight, *n* = 60 observational units; for final weight, FCR, RGR, and survival, *n* = 3 experimental units.

**Table 5:** Catalase (CAT), lipid peroxidation (LPO), total glutathione (tGSH), lysozyme, pro-phenoloxidase, and bactericidal activity levels in whiteleg shrimp (*P. vannamei*) PL fed the experimental diets for 18 days.

	NC	PC	T + M	BG	*p*-Value
CAT (U mg^−1^ protein)	22.9 ± 8.0	22.4 ± 6.3	28.9 ± 19.3	21.4 ± 7.2	0.675
LPO (nmol g wt^−1^)	14.0 ± 2.2 ^ab^	15.6 ± 3.1 ^a^	14.6 ± 2.6 ^ab^	12.7 ± 2.1 ^b^	0.039
tGSH (nmol mg protein^−1^)	4.7 ± 0.9 ^a^	5.0 ± 0.7 ^ab^	5.0 ± 0.8 ^ab^	5.7 ± 1.1 ^b^	0.018
Lysozyme (µg mg protein^−1^)	1.2 ± 0.5	1.5 ± 0.6	1.1 ± 0.3	1.2 ± 0.4	0.165
Pro-phenoloxidase (×10^−3^ U mL^−1^)	12.1 ± 6.8	14.0 ± 9.3	12.4 ± 3.8	13.0 ± 6.5	0.854
Bactericidal activity (%)	12.6 ± 6.9	12.9 ± 8.1	14.6 ± 11.9	14.5 ± 7.8	0.551

Results expressed as mean ± standard deviation (*n* = 3 experimental units). Represented are also the *p*-values for a one-way ANOVA. Different superscript letters indicate statistical differences (*p* < 0.05) between treatments in a post hoc Tukey multiple comparison test.

**Table 6:** Relative expression to housekeeping (*bactn* and *rpl-8*) of target immune related genes of whiteleg shrimp (*P. vannamei*) PL fed the experimental diets for 18 days.

Gene	Acronym	Relative Expression				*p*-Value
		NC	PC	T + M	BG	
PvHm117 crustin P	*crus*	0.6 ± 0.2 ^a^	1.1 ± 0.5 ^ab^	1.3 ± 0.4 ^b^	1.5 ± 0.8 ^b^	0.003
Penaeidin-3a	*pen-3*	0.4 ± 0.3 ^a^	1.1 ± 0.4 ^b^	0.7 ± 0.5 ^ab^	1.2 ± 0.8 ^b^	0.001
Hemocyanin	*hmc*	1.1 ± 0.7 ^b^	1.9 ± 2.4 ^ab^	0.2 ± 0.2 ^a^	0.7 ± 0.7 ^ab^	0.029
Lysozyme C-like	*lys*	0.7 ± 0.4	1.1 ± 0.5	1.3 ± 0.6	1.3 ± 0.9	0.212
C-type lectin 2-like	*lect*	0.7 ± 0.4	1.1 ± 0.5	1.3 ± 0.7	1.3 ± 1.0	0.236
Thioredoxin 1	*trd*	1.0 ± 0.5	1.0 ± 0.2	1.0 ± 0.3	0.90 ± 0.3	0.819
Glutathione transferase	*gst*	0.9 ± 0.5	0.9 ± 0.4	0.6 ± 0.3	0.4 ± 0.1	0.218
Glutathione peroxidase	*gpx*	1.0 ± 0.4	1.1 ± 0.4	0.9 ± 0.1	1.0 ± 0.3	0.622
Caspase 3	*casp-3*	0.6 ± 0.3	0.8 ± 0.4	0.6 ± 0.5	1.9 ± 2.5	0.410

Results expressed as mean ± standard deviation (*n* = 3 experimental units). Represented are also the *p*-values for a one-Way ANOVA. Different superscript letters indicate statistical differences (*p* < 0.05) between treatments in a post hoc Tukey multiple comparison test.

## Data Availability

The data presented in this study are available on request from the corresponding author. The data are not publicly available due to confidentiality issues.
